# Expression and regulation of transcript for the novel transmembrane protein Tmem182 in the adipocyte and muscle lineage

**DOI:** 10.1186/1756-0500-1-85

**Published:** 2008-09-19

**Authors:** Yu Wu, Cynthia M Smas

**Affiliations:** 1 Department of Biochemistry and Cancer Biology and Center for Diabetes and Endocrine Research, University of Toledo, Health Science Campus, Toledo, OH 43614, USA

## Abstract

**Background:**

White adipose tissue is not only an energy storage organ; it also functions as an endocrine organ. The coordination and integration of numerous gene expression events is required to establish and maintain the adipocyte phenotype.

**Findings:**

We previously observed a 45-fold upregulation for a transcript encoding a novel predicted transmembrane protein, Tmem182, upon brown preadipocyte to adipocyte conversion. Here we use real-time PCR analysis to further characterize Tmem182 transcript expression in the adipocyte lineage. Analysis across a panel of 10 murine tissues revealed highest Tmem182 transcript expression in white adipose tissues (WAT), with 10-fold to 20-fold higher levels than in brown adipose tissue (BAT). Tmem182 transcript expression is ~3-fold upregulated in BAT of genetically obese (*ob/ob*) mice *vs. *wild type C57BL/6. Analysis of three *in vitro *models of white adipogenesis indicates markedly enriched expression of Tmem182 transcript in adipocytes *vs. *preadipocytes. Compared to 3T3-L1 preadipocytes, a 157-fold higher level of Tmem182 transcript is detected at 3 day post-induction of adipogenesis and an ~2500-fold higher level in mature 3T3-L1 adipocytes. TNFα treatment of 3T3-L1 adipocytes resulted in a ~90% decrease in Tmem182 transcript level. As skeletal muscle and heart were also found to express Tmem182 transcript, we assessed expression in C2C12 myogenesis and observed a ~770-fold upregulation upon conversion of myoblasts to myocytes.

**Conclusion:**

WAT is the most prominent site of Tmem182 transcript expression and levels of transcript for Tmem182 are altered in adipose tissues of *ob/ob *mice and upon exposure of 3T3-L1 adipocytes to the proinflammatory cytokine TNFα. The dramatic upregulation of Tmem182 transcript during *in vitro *adipogenesis and myogenesis suggests Tmem182 may function in intracellular pathways important in these two cell types.

## Background

White adipose tissue (WAT) is the major site for storage of excess energy and these triglyceride stores are mobilized to meet the energy needs of the organism. Adipose tissue is now also recognized as an endocrine organ with synthesis and secretion of a variety of soluble factors such as leptin, resistin, adiponectin, retinol binding protein-4 and TNFα [1-4]. Adipocytes make up from one-third to two-thirds of the cell population found in adipose tissue, with endothelial cells, nerve cells, macrophages, fibroblast-like interstitial cells and preadipocytes, and perhaps other cell types, comprising the remaining stromal-vascular component [5]. Mature adipocytes form as the result of the differentiation of preadipocyte precursors present in adipose tissue [6-10]. Established preadipocyte cell lines such as 3T3-L1 [11] have been an extensively used *in vitro *model to define genes central to the adipocyte phenotype [12,13]. Adipogenesis is accompanied by increased transcription of genes that encode proteins key to adipocyte function, for example lipogenesis, lipolysis, lipid transport, and hormone responsiveness [7,14,15]. *In vitro *and *in vivo *studies have uncovered a pivotal role for peroxisome proliferator-activated receptor γ (PPARγ), a member of the ligand-activated steroid hormone receptor family, in the adipogenic program [8,10,16-19]. Studies have also illustrated the important contributions of the CCAAT/enhancer-binding proteins (C/EBPs) and other transcriptional signals to adipogenesis [8,10,20].

## Methods

Culture of cell lines and adipogenic conversion for 3T3-L1, ScAP-23 and wt-BAT, for the fractionation of adipose tissues, and for culture and differentiation of murine preadipocytes from subcutaneous (SC) WAT was as described [21,22]. C2C12 cells were maintained and passaged as subconfluent cultures in DMEM with10% FBS. For differentiation, cultures at 70% confluence were switched to DMEM with 2% horse serum and 10 μg/ml insulin, and were cultured under these conditions for 7 days. For treatment of 3T3-L1 adipocytes with TNFα and various pharmacological inhibitors the method was as described [23-25]. After serum-starvation for 6 h, 3T3-L1 adipocytes were pretreated with either 50 μM LY294002, 50 μM PD98059, 20 μM SB203580, 100 nM wortmannin, 1 μM rapamycin (Sigma-Aldrich, St. Louis, MO), or DMSO vehicle for 1 h and then cultured in 10 ng/ml of TNFα for 16 h in the presence of inhibitors. RNA was purified using TriZol Reagent (Invitrogen Corp.) according to manufacturer's instruction. For studies of Tmem182 transcript expression in murine tissues, 8 wk old C57BL/6 or *ob/ob *male mice were utilized, with all animal treatments conducted with approval of the University of Toledo Health Science Campus Institutional Animal Care and Use Committee.

Real-time PCR analysis was as previously described [23,24,26]. For this, total RNA was subject to purification with an RNeasy RNA purification kit with DNase I treatment (Qiagen Corp., Valencia, CA) and 5 μg used for first strand cDNA synthesis with SuperScript II RNase H-reverse transcriptase (Invitrogen Corp.) and an oligo(dT)-22 primer. Real time PCR was conducted with an ABI 7500 Real Time PCR System. Target cDNA levels were analyzed by SYBR green-based real-time PCR in 25 μl reactions containing 1× SYBR Green PCR Master Mix (Applied Biosystems, Foster City, CA), 100 nM each forward and reverse primers, and 10 ng of cDNA. Analyses were performed in triplicate and expression of each gene was normalized against Gapdh transcript level. The cycle threshold value was generated using ABI PRISM 7500 SDS software version 1.2 and exported to an Excel spreadsheet to calculate fold differences. Sequence of PCR primers used were: Tmem182 (5'-ACACCAATCAGCCACCATCC-3' and 5'-GCCACGGTAAATAATTGCGGAG-3'); Gapdh (5'-GGCAAATTCAACGGCACAG-3' and 5'-CGGAGATGATGACCCTTTTG-3'); and Myogenin (5'-GCCATCCAGTACATTGAGC-3'and 5'-GTAAGGGAGTGCAGATTGTG-3'). Primers were designed to span introns.

### Properties of Tmem182 gene and sequence

**Figure 1:**
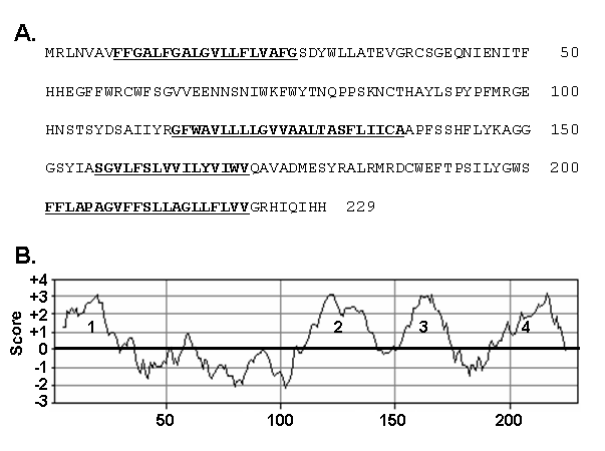
**Sequence analysis predicts a transmembrane localization for Tmem182 protein**. (A) Amino acid sequence of murine Tmem182. The numbers at right indicate amino acid positions. The four putative membrane-spanning regions are underlined. (B) Results of Kyte-Doolittle analysis with a window size of 10, indicating 4 putative transmembrane regions. Hydrophobicity score is on the *y*-axis and amino acid residue position on the *x*-axis.

During the characterization and utilization of a new *in vitro *model of brown adipogenesis, mBAP-9, we previously identified ten new genes with increased expression in mBAP-9 adipocytes *vs. *preadipocytes (manuscript in preparation). Of the ten genes identified, the gene found to exhibit the highest fold upregulation in mBAP-9 adipogenesis (45-fold), encoded an uncharacterized novel predicted transmembrane protein termed Tmem182. Its transcript expression and regulation, however, was not further characterized at the time. Tmem182 is represented by UniGene Mm.334678 [GenBank: NM_001081198]. It encodes a wholly novel 229 amino acid protein with a calculated molecular mass of 25,845. The protein sequence of Tmem182 is shown in Figure 1A. The Kyte-Doolittle analysis of Tmem182 protein sequence identifies four putative membrane-spanning regions (Figure 1B, and underlined and bolded in Figure 1A), indicative of an integral membrane topology. Database analysis (NCBI Homologene) indicates that Tmem182 homologs are found in human (90%), dog (87%), rat (87%) chick (71%), with numbers in parentheses indicating percent amino acid identity to the murine protein. Homologs are also present in pig, horse, monkey and zebrafish.

### Tissue distribution of Tmem182 transcript expression

**Figure 2:**
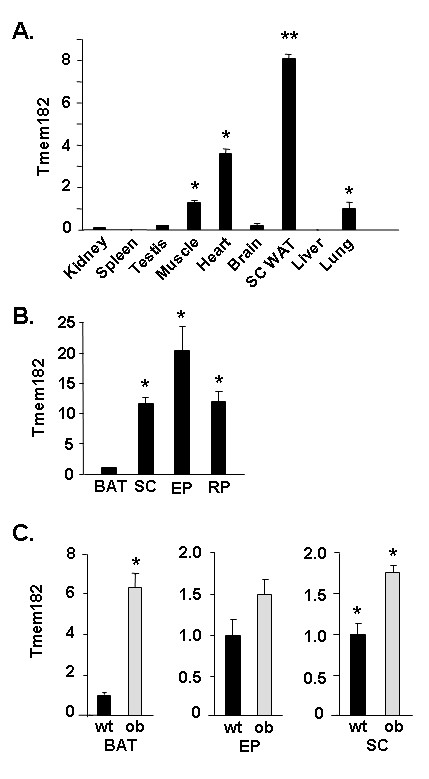
**White adipose tissue enrichment of Tmem182 and dysregulation in *ob/ob *adipose tissues**. (A) Tmem182 transcript expression in various murine tissues. RNA was harvested from the indicated murine tissues and Tmem182 transcript level was analyzed by real-time PCR as described in *Methods*. Values were normalized to Gapdh expression level and transcript expression in lung was set to a value of 1. Statistical analysis was carried out with one-way ANOVA followed by Tukey post-hoc test. ** indicates p < 0.001 for SC WAT *vs. *all other tissues. * indicates p < 0.01 for muscle, heart, and lung *vs. *all other tissues. (B) Tmem182 transcript expression in wild type brown and white adipose tissues and (C) Expression of Tmem182 transcript in wild type (wt) *vs. ob/ob *white and brown adipose tissues. RNA was harvested from subcutaneous (SC) WAT, epididymal (EP) WAT and brown adipose tissue (BAT) of wild type (wt) and *ob/ob *(ob) mice and real-time PCR for Tmem182 transcript was performed as described in *Methods*. Values were normalized to Gapdh transcript expression level. Transcript expression in BAT was set to a value of 1 for (B) and wt was set to a value of 1 for (C). Statistical analysis was carried out with single-factor ANOVA. For (B), * indicates p < 0.001 for SC, EP, and RP *vs*. BAT. For (C), * indicates p < 0.001 for ob BAT *vs. *wt BAT and for ob SC *vs. *wt SC.

We determined transcript expression of Tmem182 in a panel of murine tissues using real-time PCR. As shown in Figure 2A, of the tissues examined, subcutaneous WAT is the most enriched site of transcript expression (*p *< 0.001), with expression in muscle, heart and lung at levels from ~10% to ~50% of that in WAT. Lower relative levels of Tmem182 transcript are found in kidney, spleen, testis, brain and liver. We also examined Tmem182 transcript level in brown adipose tissue (BAT) and three distinct WAT depots, subcutaneous (SC), epididymal (EP) and retroperitoneal (RP), Figure 2B. We find that each of the three WAT depots have a similar magnitude of expression of Tmem182 transcript with from ~10- to ~20-fold higher levels (p < 0.001) than that found in BAT.

To begin to address the modulation of Tmem182 transcript levels in regard to the pathophysiology of adipocytes, we compared transcript expression in BAT and WAT from wild type C57BL/6 mice and *ob/ob *mice, the latter a well-studied murine model of genetic obesity. For Tmem182 transcript, we find a slight increase (~1.7-fold, p < 0.001) in *ob/ob *WAT for the SC depot (Figure 2C). Furthermore, compared to wt BAT, we find a 6.3-fold upregulation (p < 0.001) of Tmem182 transcript level in *ob/ob vs. *wt BAT, suggestive of a role for dysregulation of Tmem182 in the obese state (Figure 2C). It would thus appear that in the *ob/ob *genetic model, BAT shifts to a level of Tmem182 transcript expression that is more similar to that found in WAT.

### Differentiation-dependent expression of Tmem182 transcript in adipogenesis

**Figure 3:**
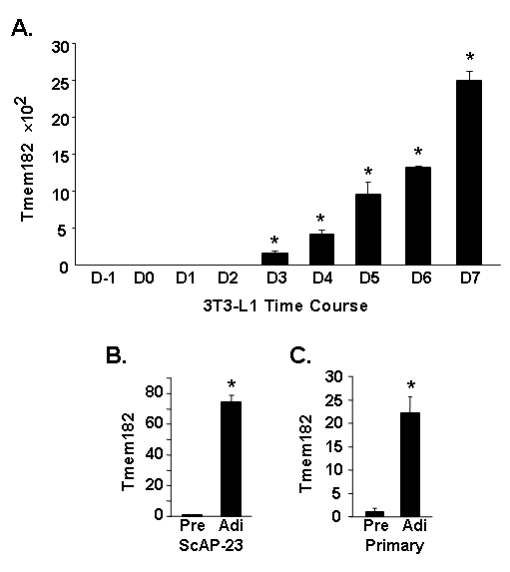
**Tmem182 is a differentiation-dependent gene in white adipogenesis**. (A) Tmem182 transcript expression during a time course of 3T3-L1 differentiation. 3T3-L1 RNA was harvested daily at the indicated days preceding (D-1) and during differentiation. Tmem182 transcript level was analyzed by real-time PCR as described in *Methods*. Values were normalized to Gapdh transcript expression level. For D-1, D0, D1, and D2, a value of 40 cycles was assigned to calculate a delta Ct value and transcript expression in D-1 sample was set to a value of 1. Statistical analysis was carried out using one-way ANOVA followed by Tukey post-hoc test; * indicates p < 0.001 for D3, D4, D5, D6 and D7 *vs. *all other samples. (B) Tmem182 transcript expression in ScAP-23 preadipocytes (Pre) and adipocytes (Adi) and (C) Tmem182 transcript expression in primary murine subcutaneous preadipocytes (Pre) and adipocytes (Adi). RNA was harvested from the indicated cultures before and after differentiation and Tmem182 transcript level was analyzed by real-time PCR as described in *Methods*. Values were normalized to Gapdh transcript expression level and transcript expression in Pre set to a value of 1. For (B) and (C), statistical analysis was carried out with single-factor ANOVA; * indicates p < 0.001 for Pre *vs. *Adi.

We originally identified Tmem182 during the characterization of a new brown adipocyte *in vitro *cell culture model, termed mBAP-9, wherein we found Tmem182 transcript to be upregulated 45-fold during the adipogenic conversion of these cells from preadipocytes to mature adipocytes (manuscript in preparation). Our tissue expression studies, however, revealed that although BAT is a site of expression of Tmem182 transcript, the most dominant site of expression *in vivo *is WAT. We therefore determined if Tmem182 transcript evidenced differentiation-dependent expression in various *in vitro *models of white adipogenesis. The best characterized cell culture model of adipogenesis is 3T3-L1, these cells were developed ~30 years ago [11] and undergo differentiation from preadipocytes to mature white adipocytes following treatment with the adipogenic agents Dex and MIX. Real-time PCR analysis indicates that 3T3-L1 adipocytes express ~2500-fold higher levels of Tmem182 transcript than 3T3-L1 preadipocytes (Figure 3A). The increase in transcript expression is first noted at day 3, with a 157-fold increase noted. In addition to the 3T3-L1 model, we examined Tmem182 transcript expression in two other models of white adipogenesis. ScAP-23 cells are a cell line established in this laboratory derived from preadipocytes present in murine SC WAT, in contrast to the embryonic derivation of 3T3-L1 cells. As shown in Figure 3B, adipogenesis of ScAP-23 cells leads to a 75-fold increase in levels of Tmem182 transcript. Figure 3C shows that adipocyte conversion of primary cultures of murine stromal vascular fraction cells from SC WAT, wherein preadipocytes are found, is accompanied by an ~22-fold increase in Tmem182 transcript. Although we find differing magnitudes of upregulation of Tmem182 transcript across the adipogenesis models that we assessed, in all cases a significant increase in Tmem182 transcript level accompanies adipocyte conversion.

### Regulation of Tmem182 transcript by TNFα

**Figure 4:**
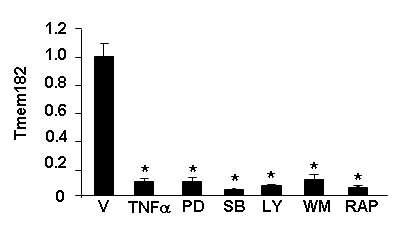
**TNFα downregulates Tmem182 transcript expression in 3T3-L1 adipocytes**. After serum-starvation for 6 h, 3T3-L1 adipocytes were incubated with either DMSO vehicle (V), 50 μM PD98059 (PD), 20 μM SB20358 (SB), 50 μM LY294002 (LY), 100 nM wortmannin (WM), or 1 μM rapamycin (Rap) for 1 h and then either treated or untreated with 10 ng/ml of TNFα for 16 h. RNA samples were harvested and Tmem182 transcript level was analyzed by real-time PCR as described in *Methods*. Values were normalized to Gapdh transcript expression level and transcript expression in vehicle alone was set to a value of 1. Statistical analysis was carried out using one-way ANOVA followed by Tukey post-hoc test; * indicates p < 0.001 for indicated samples *vs. *V alone (first column). Treatment with inhibitors alone failed to appreciably alter expression for Tmem182 transcript (data not shown).

We determined the regulation of Tmem182 transcript following incubation of in *vitro *differentiated 3T3-L1 white adipocytes with TNFα. TNFα is a proinflammatory cytokine central to adipose tissue pathophysiology and acts to suppress expression of many adipocyte genes; elevated TNFα levels are associated with obesity [27-31]. TNFα treatment of adipocytes promotes lipolysis and dedifferentiation [32-34], with the latter ascribed TNFα-mediated transcriptional downregulation of the key adipocyte transcription factors PPARγ [35] and C/EBPα [36,37]. Studies of TNFα effects on Tmem182 transcript expression were carried out using treatment with TNFα alone, or by treating cells with TNFα after adipocytes had been pretreated with pharmacological inhibitors including those for intracellular signaling pathways with a role in adipocyte gene expression and/or function. For example, reports indicate a role for p38 MAP kinase in the effects of TNFα on adipocyte gene expression [38,39] and the TNFα effects on the transcript expression of the novel adipocyte lipase ATGL are attenuated by pretreatment with PD-98059, LY-294002, or rapamycin, suggesting involvement of the p44/42 MAP kinase, PI 3-kinase, and p70 ribosomal protein S6 kinase signals [40]. As shown in Figure 4, TNFα treatment results in an ~90% decrease (*p *< 0.005) in transcript level for Tmem182 transcript. We postulated that identification of the intracellular signaling pathway(s) involved in the TNFα-mediated decrease might be one step towards gaining insights into Tmem182 function. For example, TNFα might exert its inhibitory effects by the same intracellular signaling mechanism(s) for a particular subset of genes that share similar and/or related functions in adipocytes. However, the data in Figure 4 reveal that none of the tested pharmacological inhibitors blocked the effects of TNFα, suggesting that TNFα-mediated diminution of Tmem182 transcript expression occurs *via *a signaling pathway(s) that is not reliant on p38 MAP kinase, p44/42 MAP kinase, p70 S6 or PI 3-kinase.

### Differentiation-dependent expression of Tmem182 transcript in myogenesis

**Figure 5:**
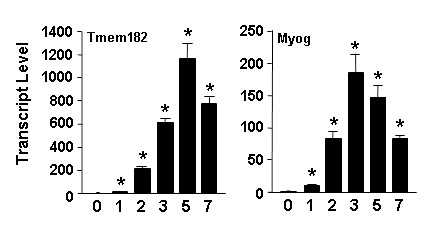
**Tmem182 is a differentiation-dependent gene in C2C12 myogenesis**. (A) Tmem182 transcript expression during a time course of C2C12 myogenic differentiation. C2C12 RNA was harvested at the time of induction of differentiation (0) or at indicated time points thereafter; numbers below graphs indicate days post-induction. Tmem182 (left panel) and myogenin (right panel) transcript levels were analyzed by real-time PCR as described in *Methods*. Values were normalized to Gapdh transcript expression level and the value in the day 0 sample set to 1. Statistical analysis was carried out using one-way ANOVA followed by Tukey post-hoc test; * indicates p < 0.001 for indicated values compared to day 0.

The tissue profiling study in Figure 2 indicates that muscle is also a site of enriched expression of Tmem182 transcript. Adipocytes and myocytes are thought to arise in development from a shared mesenchymal stem cell precursor [8,41]. The annotation for the GenBank entry [GenBank:NM_001081198] for Tmem182 indicates it was among the transcripts identified by Kuninger and coworkers in a study of novel genes induced during growth factor-mediated muscle cell survival and differentiation [42]. However, neither the regulation of Tmem182 transcript expression during myogenesis, nor any mention of Tmem182, was in their published report [42]. To determine if Tmem182 was induced during muscle differentiation we utilized the C2C12 model of *in vitro *myogenesis [43]. As is shown in the left panel of Figure 5, Tmem182 transcript is markedly upregulated during the conversion of C2C12 myoblasts to myotubes. A 10-fold increase (p < 0.001) in transcript level is noted one day post induction of differentiation and cultures at 7 days post myogenic induction express ~770 times higher (p < 0.001) levels of Tmem182 transcript compared to the level in C2C12 day 0 myoblasts. The right panel of Figure 5 shows the expression of transcript for myogenin, a muscle regulatory gene that serves as a marker for myogenesis [43,44].

### Summary

The primary amino acid sequence of Tmem182 predicts an evolutionarily conserved novel transmembrane protein. Tmem182 protein sequence lacks homologies with previously defined protein families and Tmem182 function is currently unknown. Enrichment of Tmem182 transcript in WAT, alteration in obesity, differentiation-dependent upregulation in adipogenesis and regulation by TNFα suggests that expression of Tmem182 may be integral to the adipocyte phenotype. Interestingly, Tmem182 transcript is also enriched in muscle tissue and it is markedly upregulated during *in vitro *myogenesis of C2C12 myoblasts to myocytes. This suggests Tmem182 may function in cellular pathways shared by adipocytes and myocytes but not by their respective precursor cell types. Future studies will further examine the *in vitro *and *in vivo *regulation and the function of Tmem182 in adipocytes and muscle cells.

## Abbreviations

PCR: Polymerase chain reaction; FBS: Fetal bovine serum; WAT: White adipose tissue; BAT: Brown adipose tissue; PPAR: Peroxisome proliferator-activated receptor; C/EBP: CCAAT/Enhancer binding protein; Dex: Dexamethasone; Mix: Methylisobutylxanthine; PD: PD98059; SB: SB203580; LY: LY294002; WM: Wortmannin; Rap: Rapamycin

## Competing interests

The authors declare that they have no competing interests.

## Authors' contributions

YW conducted all real-time PCR experiments. CMS carried out the analysis in Figure 1, wrote the manuscript and conceived of the study design. Both authors contributed to data analysis and interpretation, and have read and approved of the manuscript.
